# Factors contributing to the variation in antibiotic prescribing among primary health care physicians: a systematic review

**DOI:** 10.1186/s12875-023-02223-1

**Published:** 2024-01-02

**Authors:** Gashaw Enbiyale Kasse, Judy Humphries, Suzanne M. Cosh, Md Shahidul Islam

**Affiliations:** 1 School of Health, Faculty of Medicine and Health, University of New England, Armidale, 2351 Australia; 2 Department of Clinical Medicine, College of Veterinary Medicine and Animal Science, University of Gondar, Gondar, 196 Ethiopia; 3 School of Psychology, Faculty of Medicine and Health, University of New England, Armidale, 2351 Australia

**Keywords:** Antibiotic prescription, Antibiotic resistance, Physicians, Outpatient, Primary health care, Clinical decision-making

## Abstract

**Background:**

Antibiotic resistance is growing globally. The practice of health professionals when prescribing antibiotics in primary health care settings significantly impacts antibiotic resistance. Antibiotic prescription is a complex process influenced by various internal and external factors. This systematic review aims to summarize the available evidence regarding factors contributing to the variation in antibiotic prescribing among physicians in primary healthcare settings.

**Methods:**

This systematic review was conducted based on PRISMA guidelines. We included qualitative, quantitative and mixed methods studies that examined factors influencing prescription practice and variability among primary healthcare physicians. We excluded editorials, opinions, systematic reviews and studies published in languages other than English. We searched studies from electronic databases: PubMed, ProQuest Health and Medicine, Web Science, and Scopus. The quality of the included studies was appraised using the Mixed Methods Appraisal Tool (Version 2018). Narrative synthesis was employed to synthesize the result and incorporate quantitative studies.

**Results:**

Of the 1816 identified studies, 49 studies spanning 2000–2023 were eligible for review. The factors influencing antibiotic prescription practice and variability were grouped into physician-related, patient-related, and healthcare system-related factors. Clinical guidelines, previous patient experience, physician experience, colleagues’ prescribing practice, pharmaceutical pressure, time pressure, and financial considerations were found to be influencing factors of antibiotic prescribing practice. In addition, individual practice patterns, practice volume, and relationship with patients were also other factors for the variability of antibiotic prescription, especially for intra-physician prescription variability.

**Conclusion:**

Antibiotic prescription practice in primary health care is a complex practice, influenced by a combination of different factors and this may account for the variation. To address the factors that influence the variability of antibiotic prescription (intra- and inter-physician), interventions should aim to reduce diagnostic uncertainty and provide continuous medical education and training to promote patient-centred care.

**Supplementary Information:**

The online version contains supplementary material available at 10.1186/s12875-023-02223-1.

## Introduction

A pandemic of disease with antibiotic resistance is spreading throughout the planet [1]. According to the World Health Organization (WHO), approximately 80% of antibiotics are used in primary health care [2]. The use of broad-spectrum antibiotics to treat bacterial infections, especially acute respiratory tract infections, raises the risk of antibiotic resistance [3]. The practice of health professionals when prescribing antibiotics in primary healthcare settings significantly contributed to antibiotic resistance [4, 5]. The prescribing of antibiotics can vary significantly from one medical practice to another and evidence highlights that primary care physicians in different locations have variable rates for the prescription of antibiotics [6–10].

Studies reported that variation in patient characteristics, physician experience, patient expectation, power distance and the practice environment which may affect how often a physician prescribes antibiotics [9, 11]. The observed variability in antibiotic prescribing, according to Schwartz, et al. [12], may not be only explained by clinical or sociodemographic variations among the patient population. Instead, it may be related to each physician's unique prescribing patterns and patient preference. Furthermore, variance in the prescription of antibiotics by primary healthcare physicians in different nations has been observed; however, it remains unclear which factors play important roles and how different factors interact with prescribing variation [13, 14].

Published studies overlooked physicians’ beliefs on antibiotic resistance and the unforeseen factors contribute to prescription variability [15, 16]. Recent studies by Durand, et al. [17], Manne, et al. [14], and Queder, et al. [18] investigated factors influencing physician’s antibiotic prescribing practice in specific locations. These findings indicated that physicians’ antibiotic prescribing is influenced by contextual factors at the individual practice and systemic levels. However, it is 128important to note that these findings may not be generalizable to healthcare systems in other countries outside the study area. To support optimal prescribing interventions, understanding the underlying cause of variation is crucial [19, 20]. Due to knowledge gap in this area, a systematic review is required to synthesize the existing qualitative and quantitative research on primary health care physicians’ experience with prescribing antibiotics. Therefore, this review aims to identify the potential drivers that influence prescription practice and variability in antibiotic prescribing among primary healthcare physicians.

## Materials and methods

### Protocol and registration

This systematic review was conducted based on the Preferred Reporting Items for Systematic Reviews and Meta-analysis (PRISMA) 2020 guidelines [21] and followed a predefined protocol. The review protocol was registered in the International Prospective Register of Systematic Reviews (PROSPERO) CRD42023438530.

### Eligibility criteria

The reasons for variation in antibiotic prescribing among physicians in primary healthcare settings were the main focus of the review. Studies that focused on factors that influence prescribing practices and variations in antibiotic prescriptions, published in English-language peer-reviewed journals with primary healthcare physicians as participants were included. The studies were included without restriction in study methods (qualitative, quantitative and mixed methods studies). Studies that did report factors affecting antibiotic prescription practice and variation, as well as editorials, study protocols, systematic reviews, and commentary pieces, were excluded. To address the current issues influencing antibiotic prescription practices, we included studies from 2000 onwards.

### Information source and search strategy

To identify relevant studies, we conducted a comprehensive search using electronic databases: PubMed, ProQuest Health and Medicine, Web of Science, and Scopus. A combination of relevant keywords such as "antibiotics," "prescribing," "physicians," "primary health care," and "factors” were used in the search query. A 'search strategy using 'or' rather than 'and' was employed to ensure a broad search that included all relevant articles related to prescribing practice. Additionally, the Google Scholar search engine and reference lists from included articles were utilized to retrieve further relevant studies that may have been missed through the database searching process particularly to ensure that qualitative studies were not overlook. The database and search terms were finalized in consultation with a health research librarian. The search was conducted from inception to July 12, 2023. For the full search string, see Supplement file 1.

### Study selection process

All retrieved studies were imported into EndNote 20, and duplicates were removed. The selection process was carried out independently by the first author and three co-authors (MSI, JH, and SC). This process involved the review of titles and abstracts, followed by a full -text screening. Any disagreements were handled through discussion.

### Data extraction and data items

A standardized data extraction form was developed to collect relevant information from the included studies. Data extraction of included studies was performed by the first author and the other co-authors independently. We read each article (the entire manuscript) carefully and extracted the identified data elements into our collection format. Study design, country, sample size, study objectives, study population, setting, data collection methods, data analysis, study approach, major findings (i.e. factors influencing practice and variation), and recommended interventions were extracted. Intervention is not our primary objective but during analysis of the data we extracted information about possible interventions for identified factors.

### Quality assessments

The risk of bias assessment for this review was conducted using recognized tools such as the Mixed Method Appraisal Tool (version 2018). This tool is used to appraise methodological quality for different categories of studies, including qualitative, quantitative and mixed methods studies [22]. Each included study was independently assessed by four reviewers, and any discrepancies or disagreements were resolved through discussion with other reviewers.

### Data synthesis

Following qualitative data extraction, the first author and co-authors independently identified the factors influencing antibiotic prescription practice and variation. We identified different factors from included studies and categorized these factors into three main themes: physicians related factors, patients related factors, and health system related factors, based on the framework predefined by a previous study [23]. The quantitative data were qualitatively described and integrated with qualitative data [24]. Any disagreement with authors were resolved through interactive discussion. Narrative synthesis was used to synthesis quantitative studies, the general characteristic and recommended intervention from included studies. When appropriate, numerical findings were also included in the results of the review.

## Results

**Fig.1:**
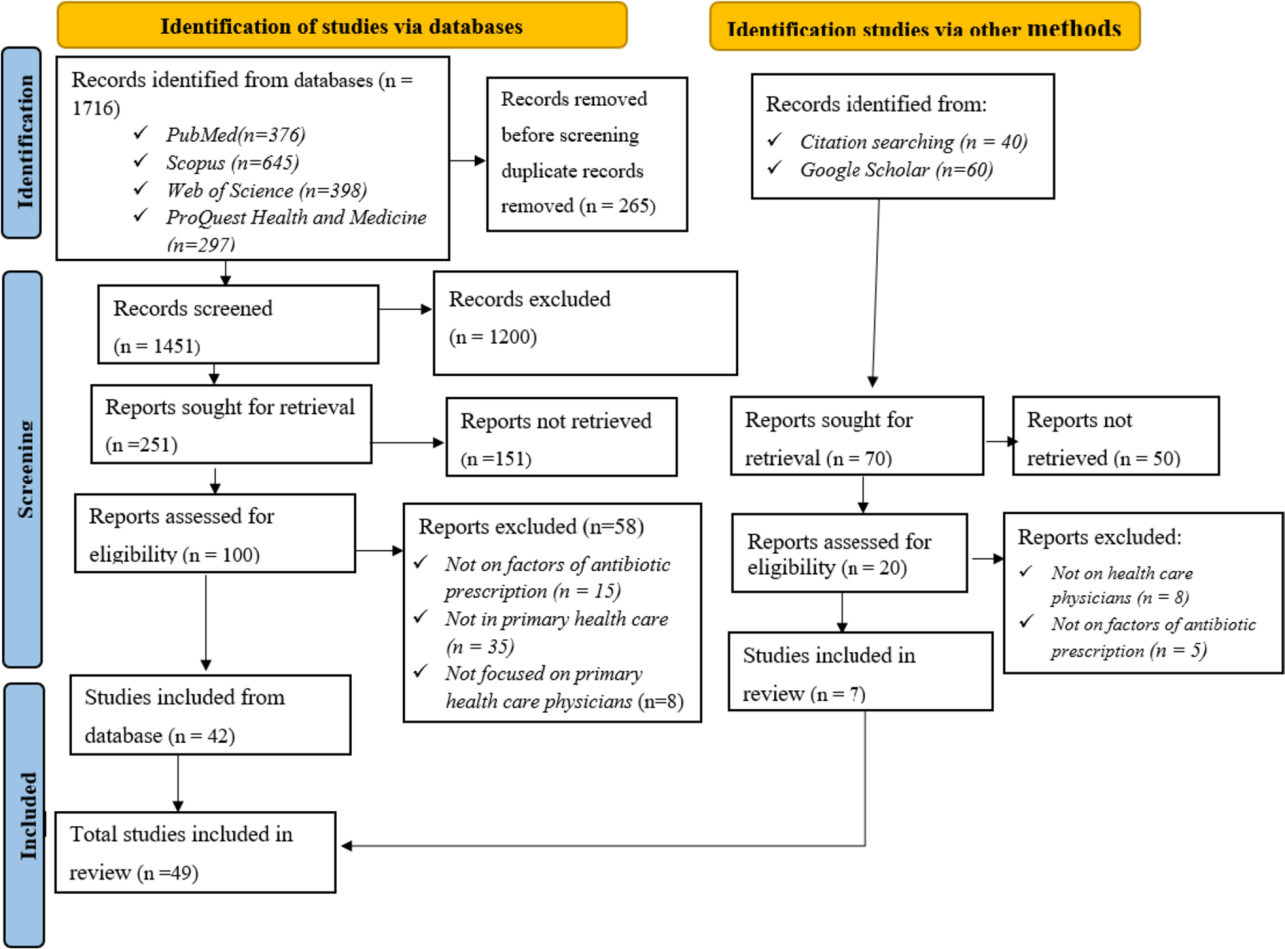
Flow diagram of study selection (PRISMA 2020 model)

A total of 1816 studies were initially identified. Of these 1816, 1716 studies were obtained from four electronic databases: PubMed (376), Scopus (645), Web of Science (*n* = 398) and ProQuest Health and Medicine (*n* = 297). One hundred studies were retrieved from Google Scholar and reference lists of the included studies. After screening titles and abstracts, 120 full-texts articles were assessed against inclusion criteria. Finally, 49 articles were included in the final analysis (see Fig. 1).

### Study characteristics

From the included studies, a total of 24 studies were conducted in European countries, four studies were conducted in Australia, and four were conducted in the USA. Additionally, three studies were carried out in China and Singapore each, with two studies each conducted in Iran, India, and Canada and one each in Cameroon, Saudi Arabia, Qatar and Myanmar. One study was carried out concomitantly in Bolivia, Paraguay and Uruguay (Table 1). From the included studies, thirty used quantitative methods, fifteen used qualitative methods and four employed mixed methods (Table 1). Regarding study design, of the 49 included studies, 47 were observational and 2 were randomized control trials. Regarding data analysis, the qualitative studies used different methods, such as grounded theory (*n* = 5) and thematic analysis (*n* = 10). The included mixed method studies used various methods: descriptive and changing point analysis (*n* = 1), framework analysis and descriptive analysis (*n* = 1), grounded theory and descriptive (*n* = 1), and narrative analysis and descriptive (*n* = 1) analysis.

**Table 1:** Study characteristics

**Author (year)**	**Country**	**Objective**	**Study design**	**Study approach**	**Study population**	**Sample size**	**Data collection methods**	**Setting**	**Data analysis**	**Overall Quality Score from 5 criteria**
Palin, et al. [25]	UK	Examine factors and variations across general practices in antibiotic prescribing	Retrospective observational study	Quantitative	Patients and physicians	587	Extract from online database	Primary health care	Descriptive and changing point analysis	5
Lum, et al. [6]	Australia	To Identify dominant factors in decision of antibiotic prescription	Observational (exploratory)	Mixed method	General practitioner	10	Interview and the discrete choice experiment	Primary health care	Thematic analysis and descriptive	5
van der Velden, et al. [20]	18 European countries	To identify Factors related to antibiotic prescription and describe between countries difference	Observational (prospective)	Quantitative	Physicians and patients	4982 patients and 143 physicians	Point-prevalence audit survey	Primary health care	Descriptive and inferential	5
Theodorou, et al. [26]	Greece and Cyprus	To investigate attitudes and the factors that influence physician prescribing decisions and practice	0bservational (cross-sectional study)	Quantitative	Physicians	1703	Survey	Both primary and secondary care	Descriptive and inferential	4
Queder, et al. [18]	Germany	To identify contextual factors associated with practitioners’ perceptions of antibiotic prescribing	Observational (prospective study)	Quantitative	Physicians	229	Survey	Primary health care	Descriptive and inferential	5
Chan, et al. [27]	Singapore	To understand the determinants influencing antibiotic prescribing decisions	Observational (cross-sectional study)	Qualitative	Physicians	19	In-depth interviews	Both secondary and primary healthcare	Descriptive and thematic analysis	4
Ahmadi and Zarei [28])	Iran	To explore the prescribing patterns and rational drug use for family physicians	observational (retrospective)	Quantitative	Family physician	184	Observation and analysing of prescriptions	Primary health care	Descriptive analysis	4
Kotwani, et al. [29]	India	To explore the factors that influence primary care physicians to prescribe antibiotics and to investigate possible interventions	Observational (cross-sectional)	Qualitative	Physicians	36	Focus group discussions	Primary healthcare	Grounded theory	4
Fletcher-Lartey, et al. [30]-	Australia	To describe the role patient expectations, play in general practitioners antibiotic prescribing	Observational (cross-sectional)	Mixed methods	Physicians	584	Survey and interviews	Primary care	Descriptive and framework analysis	5
Laka, et al. [31]	Australia	To identify perceived barriers to appropriate antibiotic prescribing across different healthcare settings	Observational (cross-sectional)	Quantitative	Physicians	180	Survey	Australian hospitals and primary care	Descriptive and content analysis	5
Swe, et al. [32]	Myanmar	To quantify prescriber variability in antibiotic prescription to patients with acute fever	Observational (retrospective)	Quantitative	Patient and physicians	1090 patient consultations with 40 doctors	From online database	Primary healthcare clinics	Descriptive and inferential	4
Mousquès, et al. [33]	France	To measure variability of antibiotic prescription by French general practitioners for acute rhinopharyngitis	Observational (retrospective)	Quantitative	Patients and physicians	778 general practitioners	Collected retrospectively from recorded patient files	Primary care and hospitals	Descriptive and inferential	4
Björnsdóttir, et al. [34]	Iceland	To map physician decision-making for common infections, exploring their diagnostics basics for antibiotic prescriptions	Observational (cross-sectional)	Qualitative	Physicians	10	Interviews	Primary health care	Ground theory	3
Bharathiraja, et al. [35]	India	To understand the antibiotic prescription pattern and factors influencing it	Observational (cross-sectional)	Quantitative	Physicians	40	Observation and analysing of prescriptions	Primary health care	Descriptive analysis	3
Cadieux, et al. [36]	Australia	To assess whether physician knowledge, time in practice place of training and practice volume explain the difference in antibiotic prescribing among physicians	Observational (retrospective)	Quantitative	Physicians	852	Extracted from online database,	Primary care physicians	Descriptive and inferential	5
Skodvin, et al. [37]	Norwegian	To investigate factors influencing doctors’ antimicrobial prescribing practices	Observational (cross-sectional)	Qualitative	Physicians	15	Interviews	Primary care and hospital setting	Thematic analysis	5
Guo, et al. [38]	Singapore	To explore processes underpinning decision-making for antibiotic prescribing by considering doctors' experience in different primary care settings	Observational (cross-sectional)	Qualitative	Physicians	30	Interviews	Primary healthcare settings	Thematic analysis	5
Borek, et al. [39]	UK	To assess social and contextual factors affecting antibiotic prescribing and engagement with antimicrobial stewardship	Observational (cross-sectional)	Qualitative	Physicians	41	Interviews	Primary health care	Thematic analysis	4
Béjean, et al. [40]	French	To identify the different practice profiles of general practitioners in order to test the hypothesis of heterogeneity in physician behaviour	Observational (retrospective)	Quantitative	Physicians	4,660	Extract from database	Primary care and hospitals	Cluster analysis and inferential analysis	5
Sydenham, et al. [41]	Denmark	To assess the importance of factors that influence decision by GPS to prescribe antibiotics for acute respiratory tract infections	Observational (cross-sectional)	Quantitative	Physicians	3336	Survey including discrete choice experiments	All healthcare settings	Descriptive and inferential	5
Schwartz, et al. [12]	Canada	To describe factors of overall antibiotic prescribing as well as the inter-physician variability in antibiotic prescribing among family physicians	Observational (retrospective)	Quantitative	Family Physicians	313	Extract from online database	Not specify	Descriptive and inferential	3
Aabenhus, et al. [8]	Denmark	To identify practice-related factors associated with high prescribers, including prescribers of critically important antibiotics as defined by WHO	Observational (retrospective)	Quantitative	Physicians	1962	Extract from online database	From all health care service	Descriptive and inferential	4
Pouwels, et al. [42]	UK	To explore to what extent factors such as patient comorbidities explain this variation in antibiotic prescribing	Observational (retrospective)	Quantitative	Physicians	348	Extract from online database	Primary health care	Descriptive and inferential	5
Paluck, et al. [43]	Canada	To investigate whether overprescribing is common in treatment of paediatric upper respiratory infections and to examine factors that influence prescribing antibiotics for antibiotic prescription	Observational (cross-sectional)	Quantitative	General and family physicians	608	Postal survey	Primary health care and family clinics	Descriptive analysis	4
Kumar, et al. [44]	UK	To understand why general practitioners, prescribe antibiotics for some cases of sore throat and to explore the factors that influence their prescribing	Observational (cross-sectional)	Qualitative	Physicians	40	Interview	Primary health care	Ground theory	3
Simpson, et al. [45]	UK	To understand of general practitioners’ perceptions of antimicrobial resistance	Observational (cross-sectional)	Qualitative	Physicians	40	Interview	Primary health care and hospitals	Grounded theory	4
Wood, et al. [46]	UK	Explore the reasons for their choice of prescribed antibiotic, in particular, their decision	Observational (cross-sectional)	Qualitative	Physicians	40	Interview	Primary health care	Grounded theory	4
Reynolds and McKee [47]	China	To assess knowledge, attitude and practice about the use of antibiotics and assess factors influencing antibiotic prescribing	Observational	Mixed methods	Patients and physicians	24 patients and 12 doctors	Survey, interviews and focus group discussion	Primary care and hospitals	Descriptive and grounded theory	4
Bjorkman, et al. [48]	Sweden	To explore and describe perception of antibiotic prescribing among Swedish hospital physicians	Observational (cross-sectional)	Mixed method	Physicians	25	Interviews and survey	Primary health care	Narrative analysis and descriptive	4
Björkman, et al. [49]	Sweden	To explore and describe the variation in GPS perception of infectious diseases management with special reference to antibiotic prescribing	Observational (cross-sectional)	Qualitative	Physicians	20	Interview	Primary care	Thematic analysis	5
Vazquez-Lago, et al. [50]	Spain	To ascertain the opinions and attitudes of general practitioners in Spain concerning antibiotics prescription and resistance	Observational (cross-sectional)	Qualitative	Physicians	33	Focus group discussion	Primary health care	Thematic analysis	4
Akkerman, et al. [51]	Netherlands	To assess determinants of antibiotic overprescribing in patients with sinusitis, tonsillitis and bronchitis in Dutch general practice	Observational (cross-sectional)	Quantitative	Physicians with their patients	146	Observation of prescribed papers	Primary health care	Descriptive and inferential	4
Wester, et al. [52]	USA	To know about prescribers' views on antibiotic resistance and impact on prescription	Observational (cross-sectional)	Quantitative	Internal medicine physicians	490	Survey	Primary health care and hospitals	Descriptive analysis	4
van der Zande, et al. [53]	UK	To understand contextual factors related to general practitioners' antibiotic prescribing behaviour in low, high, and around the mean prescribing primary	Observational (cross-sectional)	Qualitative	Physicians	41	Interviews	Primary health care	Thematic analysis	5
Chem, et al. [54]	Cameroon	To investigate prescribing patterns and predictors of antibiotic prescription in primary healthcare facilities	Observational (retrospective)	Quantitative	Physicians	30,096 prescriptions and 59 physicians	Observation of prescription and survey	Primary health care	Descriptive and inferential	4
Beilfuss, et al. [55]	USA	To estimate heterogeneous treatment responses across specialties	Randomized-controlled trial	Quantitative	Physician	645,620	Extract from recorded data and patient survey scores	Primary health care	Descriptive and inferential analysis	5
Zetts, et al. [56]	USA	To assess physicians' current attitudes towards antibiotic resistance, inappropriate antibiotic prescribing and the feasibility of outpatient stewardship efforts	Observational (cross-sectional)	Qualitative	Physicians	52	Focus group discussion	Primary health	Thematic analysis	5
Rodrigues, et al. [57]	Portugal	To compare both the attitudes and knowledge between primary care and hospital care physicians with regards to antibiotic prescribing	Observational (cross-sectional)	Quantitative	Physicians	56	Survey	Primary health care and hospital	Descriptive and inferential	4
Alradini, et al. [58]	Saudi Arabia	To identify the sociocultural and behavioural determinants that affect antibiotic prescription behaviour among primary care physicians and estimate the awareness about antibiotic resistance of public health importance	Observational (cross-sectional)	Quantitative	Physicians	434	Survey	Primary care	Descriptive and inferential	4
Tang, et al. [59]	China	To evaluate the variations in effect of public reporting in antibiotic prescribing practice among physicians with different performance in primary health care	A randomized-controlled trial	Quantitative	Physicians	60	Survey and prescription data	Primary health care	Descriptive and inferential	4
Rodrigues, et al. [60]	Portugal	To assess the influence of the determinants of physicians prescribing on the quality of antibiotic use	Observational (prospective)	Quantitative	Physicians	1094	Survey and use antibiotic prescribing quality indicators	Primary-care	Descriptive and inferential	5
Al-Homaidan and Barrimah [61]	Saud Arabia	To evaluates primary health care physicians' knowledge expectation and practice regarding antibiotics use in upper respiratory tract infections	Observational (cross-sectional)	Quantitative	Physician	294	Survey	Primary health care	Descriptive and inferential	4
Frost, et al. [62]	USA	To evaluate variation in antibiotic prescribing between paediatric and nonpediatric providers for common upper respiratory illnesses	Observational (retrospective)	Quantitative	Paediatric and physician	141 361	Extraction from online database	Primary health care	Descriptive and inferential	4
Karimi, et al. [63]	Iran	To investigate the pattern and factors affecting outpatients’ antibiotic prescribing by family physicians in primary health care	Observational (cross-sectional)	Quantitative	Family physicians	19	Evaluation of prescription pattern	Primary health care	Descriptive and inferential	5
Huang, et al. [64]	Singapore	To understand the determinant of antibiotic prescribing for URTI among junior physicians	Observational(cross-sectional)	Quantitative	Junior Physicians	130	Survey and interview	Primary health care	Descriptive and principal component analysis	5
Sharaf, et al. [65]	Qatar	Explores barriers to appropriate antibiotic prescription and pharmacists’ perspectives at primary health centres in Qatar	Observational (cross-sectional)	Qualitative	Physicians and pharmacists	50	Interview, and focus-groups discussion	Primary health care	The thematic constant comparative method	3
Poss-Doering, et al. [66]	German	To identify factors relevant to primary care physicians’ decision-making when prescribing antibiotics for acute noncomplicated infections	Observational (cross-sectional)	Qualitative	Physician	27	Interviews	Primary health care	Descriptive and thematic analysis	5
Liu, et al. [67]	China	To fill the gap, modelling physician antibiotics prescribing and identifying the potential intrinsic and external determinants of antibiotic prescribing in primary care	Observational (cross-sectional)	Quantitative	Physicians	499	Survey	Primary health care	Descriptive and two-level path analysis	5
Cordoba, et al. [68]	Bolivia, Paraguay and Uruguay	To describe and compare antibiotic prescribing patterns for primary care patients with respiratory tract infections in South American countries	Observational (prospective)	Quantitative	Physician	171	Survey and observation of their prescription	Primary health care	Descriptive and inferential	4

### Quality of studies

The quality of the included studies was assessed using the MMAT [22]. Of the total studies, twenty-one were five stars (qualified for each of the five criteria), twenty-three were four stars, and the remaining five were three stars (see Table 1). More detailed information regarding the rating of studies is available in Supplementary 2. The majority of studies that used descriptive cross-sectional methodologies applied sampling techniques that were pertinent to the study’s research question. In two mixed methods studies [47, 48], the divergences and inconsistencies between qualitative and qualitative results, along with the outcome of their integration, were not appropriately addressed.

### Synthesis of results

We have identified various factors from the included studies that influence antibiotic prescribing practices and contributed to the variation in prescribing (as shown Table 2). As explained in the data synthesis section above, we categorized the factors into three main categories: patient-related factors (knowledge, preference, expectations, culture, economic status, and previous experience), physician-related factors (expertise, knowledge, specific prescription patterns, time constraints, and communication with patients), and health system-related factors (financial incentives, guidelines, policies, and regulations).

**Table 2:** Main findings of the included studies

**Author (year)**	**Main Finding**	**Recommended intervention**
Palin, et al. [25]	Significant variability in antibiotic prescribing between practice and within practices was observed. This variability is influenced by patient characteristics which play a role in shaping antibiotic decisions	Prescribing guidelines, and more targeted interventions are needed
Lum, et al. [6]	The main challenges to prudent antibiotic prescribing are patient expectations, colleagues' prescribing habits, cultural norms, and professional routines; and uncertainty of diagnosis coupled with prescribing pressure of patients	Upskilling physicians to manage patient’s expectations efficaciously
van der Velden, et al. [20]	Point-of-care testing can confidently overturn judgements made to prescribe antibiotics solely based on clinical criteria, it could improve the quality of antibiotic prescribing decisions	The Point Prevalence Audit Survey is regarded as an important research tool, and the country-specific data it contains can help in developing and putting into practice antibiotic management initiatives
Theodorou, et al. [26]	Source of information, cost of drugs, clinical effectiveness, attitudes of physicians towards generic prescribing and innovation, and patient preference are important factors for Prescribing behaviour of physicians	When formulating policies to help physicians make better decisions and, as a result, improve clinical and financial effectiveness and efficiency, the health care system should place particular emphasis on the attitudes and factors
Queder, et al. [18]	The context of physicians' practices, the length of their work experience, and system-level influences have been identified as significant influences on their perceptions of antibiotic prescribing	Intervention studies must be conducted at a large scale to adequately investigate the diverse environment around physicians' practices
Chan, et al. [27]	Time pressures and patient demands can influence physicians' antibiotic prescribing decisions based on organizational practice norms	Patient education targeting at individual, interpersonal and community levels, could reduce unnecessary antibiotic use
Ahmadi and Zarei [28])	Perception and knowledge of physicians, the socioeconomic characteristics of patients, and the pattern of the disease can lead to irrational drug prescribing among family physicians	Training on rational use of antibiotics, and continuing education for physicians
Kotwani, et al. [29]	Important factors identified for antibiotic prescription by physicians were perceived demand and expectation from the patient, diagnostic uncertainty, practice sustainability, financial consideration, influence from medical representatives and inadequate knowledge	To encourage sensible use of antibiotics in the community, interventions such as continuing medical education for doctors, patient education, shared decision-making, and stronger laws and regulations were proposed
Fletcher-Lartey, et al. [30]-	Many doctors did not believe that the use of antibiotics in primary care was the cause of the rise in antibiotic resistance, nor did they believe that their own prescribing would have much impact given other, more important problems, such as hospital prescribing	There is a need to increase awareness of the scope and magnitude of the role primary care prescribing plays, antibiotics resistance and the contribution of individual prescribing decisions to the problems of antibiotics
Laka, et al. [31]	The use of guidelines, years of experience, and type of setting were factors of antibiotic prescribing	Designing targeted and tailored interventions for appropriate antibiotic prescribing and promote rational antibiotic prescribing practices in primary care practice and hospital settings
Swe, et al. [32]	The inter-prescriber heterogeneity in antibiotic prescribing decisions was found to be significantly influenced by several patient variables, including past antibiotic use, patient age, clinical context, and management	When developing trials and stewardship programs intended to lessen unnecessary antibiotic prescriptions, intra-prescriber variance should be considered
Mousquès, et al. [33]	Only 6% of the overall variation was related to inter-physician variability, with intra-physician variability accounting for a considerable portion (70%). Differences in early medical education, continuing medical education and more broadly the sort of medical information transmission may have an impact	Policymakers should consider the variability of antibiotic prescription among physicians in primary health care settings to develop facilitators for promoting better use of antibiotics when it is interested
Björnsdóttir, et al. [34]	The diagnostic techniques used by the doctors were very variable and individual, in contrast to being consistent throughout time. Physicians' professional experience, clinical guidelines, continuing education, and patient presentation are some of the causes for the contrast between individual variability and consistency over time in diagnostic procedures. This difference has an ongoing impact on doctor practises' decisions to prescribe antibiotics	General practitioners may need to modify their diagnostic strategies in light of new knowledge and technologies because the medical sector is continually changing
Bharathiraja, et al. [35]	Factors like experience of physician, postgraduate qualification, source and method of updating knowledge, inpatient practice setting and clinical symptoms influenced the antibiotic prescription	The task of raising doctors' understanding of the usage of antibiotics should be taken on by professional organizations
Cadieux, et al. [36]	Doctors with busy practises, and practitioners with less experience were more likely to give antibiotic prescriptions that were not necessary	More understanding of the mechanisms behind these factors of incorrect antibiotic prescribing will be necessary to create effective therapies
Skodvin, et al. [37]	Key factors influencing antimicrobial prescribing practises were identified as patient assessment, informal training by experienced colleagues, and infectious diseases specialities replacing managers in promoting prudent prescribing policies	Before developing sustainable and tailored antimicrobial stewardship programs interventions may first identify important stakeholders and organizational obstacles
Guo, et al. [38]	Financial factors, drug formulary management, patient load, and a strong patient‒physician relationship were shown to be crucial for effective antibiotic prescribing. multiple factors influencing antibiotic prescribing in primary care	Reduced inappropriate prescribing practices can be achieved in part by implementing shared decision-making in primary healthcare settings
Borek, et al. [39]	Antibiotic prescribing and engagement with AMS in primary healthcare settings in England are influenced by social and contextual factors on multiple levels (individual, local, practice, and national	It is critical to shift the emphasis towards giving prescribers, practises, and commissioners more assistance in their initiatives to enhance antibiotic prescribing practices
Béjean, et al. [40]	The scope and degree of medical intervention are influenced by patient features, individual circumstances, and the socioeconomic environment. The type of compensation, competitive environments, and financial incentives, on the other hand, have a higher impact on physician activity	To understand physicians’ behaviour and response to policy incentives, policymakers should consider the variety in physicians' practice patterns
Sydenham, et al. [41]	Creative protein level, general condition-guided, generalists, stethoscope-guided, reluctant prescribers, and are influenced factors to prescribe antibiotics for acute respiratory tract infection	In the fight against antibiotic resistance, the use of CRP testing is crucial to promote reasonable antibiotic use
Schwartz, et al. [12]	Significant inter-physician variation exists in the prescription of antibiotics in primary health care settings. Patient features could not account for this variation	The inter-physician diversity of family physicians should be considered in innervations
Aabenhus, et al. [8]	Higher prescribers of antibiotics in Danish general practice can be identified by organisational and diagnostic variables. These variables could be the size of the practice, the accessibility of prescription guidelines or protocols for antibiotics, the existence of quality improvement projects, and the degree of cooperation amongst healthcare professionals working for the same company	There is a constant demand for the general practice sector to seek to decrease the overuse of antibiotics
Pouwels, et al. [42]	Variations in comorbidity prevalence cannot account for the majority of practice-level variation in antimicrobial prescribing. The possibility for a practice to minimise prescribing may be determined by considering factors like high consultation rate for acute respiratory tract infection and higher prescription rate for corticosteroids, which may explain a large portion of the difference	Provide targeted education and training programs for healthcare providers on evidence-based guidelines for appropriate antibiotic prescribing
Paluck, et al. [43]	Perceived pressure from parents was identified by physicians as a major factor in antibiotic prescribing in this survey	A comprehensive strategy and demand that the general public be educated on current upper respiratory tract infection treatment concepts and antimicrobial medication resistance
Kumar, et al. [44]	The doctor-patient relationship, biomedical evidence, policy statements, clinical experience, social context, service provision and individual knowledge of patients were a given factor that led to prescribe antibiotics	Implement electronic clinical decision support systems within electronic health records
Simpson, et al. [45]	Updating guidelines on antibiotic prescribing for physicians is crucial to emphasize the significance of appropriate antibiotic use in containing the problem of antimicrobial resistance	Develop multifaceted therapies that target both diagnostic and operational issues by integrating educational programmes, clinical decision assistance, and system-level adjustments
Wood, et al. [46]	The primary factors for prescribing antibiotics were clinical considerations such as the presenting conditions, patient circumstance, the perceived need to treat the infection immediately and effectively, the likely infecting organisms, perceptions of resistance and treatment failure, a duty to provide patients with the opportunity to benefit and likelihood of re-presentation	The strategies to change broad-spectrum antibiotic prescribing will need to consider clinicians’ perceptions of social responsibility
Reynolds and McKee [47]	The existence of financial incentives and the limited availability of information on appropriate prescribing, Perceptions of antibiotic resistance, knowledge gap, and inadequate guidance are the main factors for inappropriate of antibiotics	A multifaceted approach that includes increased surveillance, harmful incentives being replaced with ones that encourage best practise, and education based on an understanding of existing attitudes
Bjorkman, et al. [48]	The prudent antibiotic prescription was hindered by the patient's caretaking priority, a lack of attention on restricted antibiotic use, a lack of knowledge about how to treat infectious infections, or pressure from the healthcare organization	Collaboration across disciplines; for effective antibiotic resistance prevention, cooperation amongst healthcare experts is necessary
Björkman, et al. [49]	Restrictive antibiotic prescribing was considered crucial to combat antibiotic resistance, although the actual prescribing was greatly influenced by the interaction between patients and physicians	Training in communication skills is essential for physicians and other healthcare workers
Vazquez-Lago, et al. [50]	Complacency, fear, patient’s insufficient knowledge and external responsibility of the pharmaceutical and over-the-counter antibiotics were the factors that influenced the prescription of antibiotics by general practitioners	No
Akkerman, et al. [51]	In daily practice, it has been noted that physicians can overestimate the severity of symptoms and might think that patients have higher expectations when considering whether to prescribe antibiotics for respiratory tract infections. This propensity may lead to the misuse of antibiotics in cases where they may not be required or effective	Using patient-centred counselling techniques to justify the prescription of antibiotics
Wester, et al. [52]	The improvement of antibiotics prescribing and infection control practises may be restricted by disparities in physician knowledge, beliefs, and attitudes. Encouraging the use of alternative therapies, putting guidelines into place, and making sure doctors provide patients with the right advice, it is critical to overcoming disparities	The treatments that provide knowledge without influencing clinicians' actions were the most appreciated. These interventions included giving out up-to-date antibiograms, administering antibiotics in accordance with institution-specific prescription guidelines, and holding grand rounds on antibiotic prescribing and antibiotic resistance
van der Zande, et al. [53]	Experience and confidence in clinical decision-making are crucial factors in addition to acknowledging patient concerns and reaching shared decisions during consultation. However, time pressure, especially time availability for consultation can have an impact on increased antibiotic prescribing in primary healthcare. During the decision-making process, effective communication between doctors and patients, as well as addressing patients' expectations, significantly influences antibiotic prescribing	Population-level initiatives and clinician-led programs have demonstrated the efficacy of communication-based interventions directed at the general public in lowering antibiotic prescribing
Chem, et al. [54]	The socioeconomic status of patients in public health facilities, the drug availability in healthcare facilities, and the in-service training of prescriptions in private healthcare facilities were all recognised as factors that affect antibiotic prescription	Performance Based Financing scheme should apply in primary health care settings and prescribing should only be done by physicians as they have adequate training
Beilfuss, et al. [55]	Understanding physician prescribing behaviour for antibiotics requires consideration of aspects such as physician affiliations, characteristics, quantity of treatment, and patient characteristics Additionally, the use of antibiotics by doctors has been measurably affected by physician-based policy	Promote effective medical service delivery and high-quality care through better physician coordination and accountability
Zetts, et al. [56]	Patient demand, physician perceptions of broader quality measurement systems, financial incentives, patients' past experience of receiving antibiotics from another clinician, physicians’ belief that antibiotic knowledge deficits were key drivers of overprescribing	The intervention for Antibiotic Stewardship Program should consider physician attitudes and beliefs about antibiotic stewardship
Rodrigues, et al. [57]	The difference in opinion between physicians working in hospitals and primary care can have impact on the quality of antimicrobial prescribing. These disparities arise due to patient volume, specialist expertise, treatment guidelines and practice and resource availability	The intervention to improve antibiotic prescription quality should be costumed for each setting, especially considering the more evident difference between primary care and hospital attitudes
Alradini, et al. [58]	Professional status, Workplace, and duration of clinical practice were factors prescription of antibiotics by primary health care physicians. The senior and more clinical experienced physicians with higher professional degrees had higher control towards antibiotic prescription	Regular conferences, workshops, and continuing medical education are used to train physicians to expand their knowledge
Tang, et al. [59]	Public reporting can positively influence antimicrobial prescribing patterns of doctors particularly for acute respiratory tract infections in primary health care settings, with reduction in the prescription rate of antibiotics and use of antibiotics	Public reporting intervention with special concentration on the physician prescription patterns
Rodrigues, et al. [60]	Working in the emergency department, workload, and physicians’ attitudes were identified as critical factors affecting antibiotics prescriptions	Junior doctors' knowledge and clinical behaviour should be improved, and decision-makers should be made aware of the connection between higher workload and subpar performance when prescribing antibiotics
Al-Homaidan and Barrimah [61]	Physicians have some shortage of knowledge and attitude about antibiotics regarding beneficial effects of antibiotics, the efficacy of alternatives to antibiotics, antibiotic resistance, harmful effects of antibiotics, practice guidelines, and the advice that should be given to patients who are prescribed antibiotics	The adoption of practice guidelines, bettering patient awareness and education, and rules for prescription and dispensing antibiotics are some of the more targeted treatments that primary healthcare providers need
Frost, et al. [62]	Knowledge deficits regarding current guidelines, specialities and peer prescribing habits, experience, confidence level treating patient and parent factors were main barriers for antibiotic prescription. Additionally, diagnostic uncertainty is a leading driver of antibiotic prescribing	To focus Antibiotic Stewardship Program efforts; research on the knowledge, attitudes, and beliefs influencing prescribing practices can be useful
Karimi, et al. [63]	Study of experience, cultural and societal characteristics, and belief in the significant impact of antibiotic prescription. Self-medication, as well as the people's habits and cultural elements as a whole, encourage them to recommend doctors who frequently prescribe antibiotics	There is a need to increase family doctors' knowledge and proficiency in prescription antibiotics in the primary healthcare setting
Huang, et al. [64]	Diagnostic uncertainty and knowledge gaps, Organizational-related factors (organization norms and culture) were determinants of antibiotics prescribing practices	Antibiotic prescribing can be made more effective by modelling institutional best practice standards and clinical decision support systems based on local epidemiology
Sharaf, et al. [65]	Practitioners mainly physicians, patients and the organizations themselves played a role in shaping antibiotic uses and prescribing practice in primary healthcare centres. Patient’s behaviour, patient pressure, workload and restricted time of consultation and management response to patient complaints were strong factors of antibiotic prescribing practices	Effective behavioural change initiatives should consider a variety of elements, including individual and organizational aspects
Poss-Doering, et al. [66]	Continuity of care, patient expectations, uncertainty regarding diagnosis, prognosis, and when not knowing the patient are main factors in physicians' developed habits in decision-making on antibiotics prescribing	No
Liu, et al. [67]	Antimicrobial prescribing practices are complex processes and associated with external factors; financial incentives, patient pressure and time pressure) and intrinsic regarding prescriber (knowledge and attitude)	It is crucial to implement policy initiatives that focus on external issues connected to the prescription of antibiotics
Cordoba, et al. [68]	The variability of antibiotic prescription is explained by diagnostic uncertainty and contextual characteristics beyond clinical practice	Providing physicians with evidence-based guidelines and tools to apply them

#### Factors influencing antibiotics prescribing practice

Factors influencing prescribing practice include patient-related factors, health system-related factors, and health system related factors.

##### Patient related factors

Some of the included studies mentioned that the practitioner’s perception of patients influences the antibiotic prescribing practise of physicians [6, 31, 36–38, 43, 58, 69, 70]. A study conducted in China reported that patients often had an expectation of receiving antibiotics when they visited healthcare providers, and a perception of this expectation pressures physicians to prescribe [47]. Another study [27] noted that physicians frequently overprescribe antibiotics due to patient expectations and preferences. The expectation and preference of patients who have previously received antibiotics have an effect on the decision of physicians [12, 32, 34, 42, 52].

Reynolds and McKee [47] noted that the economic status of patients affects both the actual and perceived need for antibiotics. Another study also reported that patients do not want to waste money on another prescription; instead, they use their previous prescription to purchase drugs from private or public pharmacy [28, 40]. Similarly, a study focused on prescribing patterns and factors associated with antibiotic prescription in primary healthcare facilities reported that patients with lower financial capacity exerted less pressure on physicians to prescribe antibiotics [54]. In contrast, two studies reported that patients with low income, individual unemployment status and high uncertainty avoidance (minimize risk and unpredictability) are likely to result in patient demand for physician prescription of antibiotics [29, 31] (as shown Table 2).

Patient social characteristics such as education, health literacy levels, and occupation have potentially impacted the perceived need for prescribing antibiotics [25, 27, 50]. Cultural views on health and illness, attitudes about health, and causes of diseases are other patient characteristics that influence antibiotic use. A study conducted in the UK reported that patients seeking immediate relief, cultural beliefs and their previous experience with specific drugs contributed to variability in the prescribing practice of physicians [46]. Another study conducted in India reported that patients who engage in self-diagnosis and self-medication requested specific antibiotics compared with patients who have no knowledge about antibiotics [29]. In addition, the study also found that the patient’s relationship with physicians and the deliberate exaggeration or misinformation of symptoms affect the prescription of antibiotics [38, 51].

##### Physicians-related factors

Studies show that physicians who actively refresh their expertise through continuing medical training, workshops, seminars and journals, are less likely to prescribe antibiotics [35, 65, 66]. Another important factor constantly reported through the study is time pressures [27, 30, 65, 67]. Two additional studies also reported that diagnostic uncertainty combined with time constraints influenced physicians’ antibiotic prescription practice [8, 39]. A study conducted in the UK reported that time pressure, especially the limited time available for consultation, has an impact on increased antibiotic prescription in primary health care [53]. Chem, et al. [54] reported that complacency, fear, and insufficient knowledge were other factors that influenced the prescribing of antibiotics by physicians. Kumar, et al. [44] and Karimi, et al. [63] stated that knowledge, skills and insights acquired through interaction with patients and medical cases were associated with physicians’ prescription practices and decisions to prescribe antibiotics.

On the other hand, physicians who only practice outpatient medicine are more likely to prescribe antibiotics than in patients [36, 54, 68]. Many physicians did not think that antibiotic prescribing in primary care was responsible for the growth of antibiotic resistance or that their individual prescribing could make any difference in light of other issues, such as hospital prescribing [45, 49, 52]. The included studies show that engagement with antibiotic stewardship in primary health care influences prescribing by reducing the frequency of prescription [6, 28, 38, 63].

##### Health system-related factors

In this review, studies reported that organizational-related factors influenced the antibiotic prescribing practice of physicians. The studies conducted by Sharaf, et al. [65], Liu, et al. [67] and Poss-Doering, et al. [66] reported that antibiotic prescribing is affected by the presence of and adherence to evidence-based clinical guidelines and protocols for antibiotic use. Quantity and quality of service were also associated with antibiotic prescribing practices and variability of prescribing antibiotics in primary health care physicians [46, 58]. Weak regulation of health systems, the dissemination of medical information, and practice setting characteristics (such as location, level of activities, network participation, and continuing medical education) influenced antibiotic prescribing [30, 32, 33, 47, 58]. Chem, et al. [54] reported that the external pressure of the pharmaceutical industry and over-the-counter antibiotics were factors that influenced the prescribing of antibiotics by physicians. Two other studies reported that biomedical evidence, policy statements and service provision were factors influencing prescribing [44, 63].

#### Factors for intra- and inter-physician variability in antibiotic prescription

##### Physicians-related factors

Factors contributing to intra-and inter-physician variability in antibiotic prescriptions are physician’s related factors, such as physicians’ expertise, knowledge, attitudes, beliefs, clinical experience, and particular prescription practices. A study conducted in the UK reported that there was large variability in antibiotic prescriptions between and within health care providers for the same condition [25]. Another study also reported that the variation in antibiotic prescriptions in primary health care was due to intra-physician variability (70%), with only 6% due to inter- physician variability [33]. This variation was largely explained by patient characteristics and practice setting characteristics (location, level of activity, continued medical education and network participation) (as shown Table 2). However, a study conducted in Canada reported that the inter-physician variability in prescribing antibiotics could not be explained by patient preference and patient sociodemographic characteristics; rather, it is likely to be related to individual physicians’ prescribing habits [12]. Clinical setting and management were also important factors for inter-prescribing variability in the decision to prescribe antibiotics [12, 32, 34, 42, 52].

Physician affiliations (institutional characteristics) are important factors in understanding the variation in physician antibiotic prescribing practices [58, 71]. Loss of control over prescribing decisions, evidence-based practice, and differences in priorities among different doctors are some of the factors that influence the variability of prescription [27, 29, 49, 50, 55]. More experienced physicians prescribe fewer antibiotics than junior physicians in regular clinical work, particularly during times of uncertainty [8, 37]. Another study conducted in the USA also reported that physicians with higher qualifications (specialties, higher level of expertise) and those with more experience were less likely to prescribe antibiotics [62]. This study also reported that specialized paediatric physicians were more likely to adhere to guidelines for managing the treatment and less likely to prescribe antibiotics without positive tests. The included studies also showed that the majority of practice-level variation in antibiotic prescribing was explained by the variation in physicians’ individual practice patterns, perceptions, attitudes, and knowledge [12, 18, 32, 42, 43] (as shown in Table 2). According to Tang, et al. [59], exposure to different medical cases can have a favourable impact on the variability of antibiotic prescribing patterns of physicians for upper respiratory tract infections. Additionally, interpretations of symptoms, workload, and working at emergency departments lead to variations in prescribing practices [12, 60]. Bharathiraja, et al. [35] explained that factors such as interactions, discussions and exposure to different prescribing behaviours with colleagues and inpatient practice settings influenced antibiotic prescription behaviours.

## Discussion

The objective of this study was to provide an overall picture of the current published evidence of factors of antibiotic prescription and variability thereof in primary health care physicians. Various factors could be identified that might influence prescribing practices and contribute to variability in antibiotic prescribing. For a more in-depth understanding, the theses factors are discussed in more detail within the following major themes.

### Patient-related factors

In this review, we found that the influence of patients on antibiotic prescription practice emerged as a noteworthy and significant finding. There is evidence that patient attitude, knowledge, or beliefs had an influence on antibiotic prescription in primary healthcare physicians, for example, misconceptions regarding the role of antibiotics regarding the efficacy of antibiotics in treating viral infections and infectious diseases [25, 31, 41, 46, 60, 72]. This finding is supported by a study conducted in Canada that revealed an increase in antibiotic use by patients during the influenza season [73]. Furthermore, this review identified that the perception of what is considered concerning symptoms, the perceived need to consult a physician, and faith in the body's natural healing power, have an impact on antibiotic use [12, 72, 74, 75].

Another piece of evidence we found in this review pertained to the habits and cultural factors of the patients can encourage frequent prescribing and self-medication [63, 64]. This might be the result of cultural norms and traditions may shape individual views on health, favouring for quick relief through antibiotics. These cultural norms and traditions may be influenced by patients’ previous experience with obtaining treatment from other clinicians [36, 56, 76, 77]. Another important point from this review is that the socioeconomic status of patients was all recognized as factors that affect antibiotic prescription [40, 54]. Patients with a high economic status might exert more pressure on physicians to prescribe antibiotics than patients with lower economic status [29, 38, 77, 78].

### Physician-related factors

In this review, we found that physicians’ attitudes about antibiotic resistance and prescribing habits influence antibiotic prescribing [30, 46, 74, 79]. A lack of understanding of antibiotic resistance may contribute to the variation in antibiotic prescription. Updating guidelines for physicians on antibiotic prescribing is crucial to emphasize the significance of appropriate antibiotic use in addressing antibiotic resistance issue [45]. Furthermore, clinical experience, such as exposure to infectious disease, influences antibiotic prescription practice [44]. This implies that physicians with more clinical experience and knowledge of Antibiotic stewardship programs tend to prescribe antibiotics less frequently. This is also supported by another study [53], which shows that physicians with high experience in diagnosing and treating various medical conditions exhibited lower prescribing due to their heightened confidence. According to Queder, et al. [18], the physician's attitude toward sustainable use of antibiotics is based on professional experience in prescribing and acquired knowledge about antibiotics. This suggestion is supported by another study [43] junior physicians might be more likely to be guideline oriented than senior physicians.

According to three studies, physicians with high practice volumes are more likely to prescribe antibiotics inappropriately or excessively compared to those with low practice volumes [36, 80, 81]. This indicated that busy physicians’ pressure to treat many patients in a short period of time may make generalized diagnoses, resulting in antibiotic prescribing even when it is not necessary. This aligns with studies indicating physicians prescribe antibiotics unnecessarily to expedite clinic visits and improve patient satisfaction [37, 82, 83]. Physician perception of patients’ expectations of antibiotics are other factors that influence prescribing practice [6, 49]. This finding is also supported by another study conducted in India, where many physicians perceived that patients expect them to prescribe antibiotics after spending money on consultation, which leads to dissatisfaction if antibiotics are not provided. This review notes prescribers are influenced by the desire for positive patient relationships [29, 38, 44, 49, 53]. This indicates that physicians may assume that patients want antibiotics to boost satisfaction [84–86]. According to Schwartz, et al. [12], family physicians show significant variability in antibiotic prescribing not entirely explained by patients’ characteristics. This might be due to the financial incentive for prescribers, and lack of continuous medical education.

In this review, we found that antibiotic prescription practices are highly influenced by medical colleagues’ prescribing behaviour and conduct [6, 37]. A previous systematic review noted that physicians often share insights, and seek advice from their colleagues, which may shape their approach to antibiotic prescription [87]. Studies from Ireland and the UK reported that a hierarchical system, particularly senior colleagues, influenced physicians’ antibiotic prescribing practices [88, 89]. These hieratical influence can significantly shape physician prescribing decisions but misunderstandings of the responsibilities and roles pose obstacles to antibiotic prescription.

#### Health system-related factors

Another factor found in this review shows that antibiotic prescribing can vary significantly based on the resources available, financial capacity, and regulation of the healthcare setting. In a setting where formal guidelines are lacking regarding antibiotic prescriptions, physicians often rely on their individual knowledge and previous experience, which may result in over prescription or inadequate use of antibiotics [47, 64, 90]. According to Harbarth and Samore [91], clinical guidelines that specifically tailored to the situation, governing over-the-counter prescription of antibiotics, resulted in reduced antibiotic use. In this review, we found that healthcare system norms and culture significant influence antibiotics prescribing [62, 64]. The setting that prioritizes communication and encourages discussion can lead to more precise and targeted antibiotic prescription practices. Ness, et al. [92] and Skodvin, et al. [37] noted that financial incentives and healthcare regulations influence antibiotic prescription. A study conducted in Japan reported that financial incentives to medical facilities for not prescribing antibiotics resulted in reduced antibiotic prescribing [93].

### Interventions to address major factors influencing variation of antibiotic prescription

This review found that the variation in antibiotic prescribing practice was due to intra- and inter-physician variability in response to factors related to patients, physicians and health system. This review presents major factors that could be targeted for developing interventions (Table 3).

**Table 3:** Summary of major identified factors with potential intervention

**Factors**	**Description**	**Example of potential recommended intervention**
Diagnostic uncertainty	Lack of confidence about diagnosis	Providing education on dealing with uncertainty in medical practice Promoting rapid diagnostics e.g. Applying point-of-care testing [31, 64, 68]
Physician‒patient relationship	The communication between patients and physicians during consultation	Improving communication skills and public knowledge of antibiotics through antibiotics awareness campaigns, and implementing shared decision-making [38, 44]
Guidelines	Attitudes towards practice guidelines and beliefs on applicability of guidelines	Improving applicability of guidelines and letting prescribers participate in hospital guideline formation to improve, support and adherence [8, 25, 42, 45, 61]
Clinical experience and education of physicians	Prior individual cases, types of clinical experience, years and education	Facilitating training for junior and young professionals, on antibiotic prescription and stewardship [44, 58]
Source of updating knowledge/source of information	Up-to-date knowledge and reliable sources of information	Facilitating accessibility of online databases, continuing medical education and guidelines from health organizations [26, 35, 57]
Colleagues' pressure and prescribing habits, and professional routines	Colleagues and senior physicians in the decision process for prescribing antibiotics	Educating senior physicians about their position as role models and teaching them how to explain why they are using antibiotics. improving young physicians' ability to reflect on their superiors' antibiotic prescriptions [6, 37]
Financial factors	Finance incentives, access to and investments in healthcare	Continuous monitoring of the payment system of health care providers [38, 40, 47]
Patients’ previous experiences of receiving antibiotics and cultural perceptions of illness and health	The history of antibiotic use and beliefs about health, cause of diseases and labelling of illness	Patient education, communication skill training and implementing shared decision-making [56]
Attitude, perception and knowledge of physicians regarding antibiotic resistance	The physician responsible for growing antibiotic resistance and awareness of it	Promoting feedback to physicians on their own prescription characteristics and distributing posters/leaflets to increase awareness about antibiotic resistance [30, 48]
Practice volume and time pressure	Number of patients who physicians manage within specific time	Optimize appointment scheduling, providing telemedicine service and optimization of workflow [32]
Demographic and social determinants of patient health	Poverty, low education level, living place, comorbid conditions, access to health care	Implement income support programs like health insurance, promoting health literacy and education programs [55, 94–97]
Over-the-counter prescription of antibiotics and pressures from pharmaceutical industries	Purchased without a prescription from pharmacies and pressure from pharmaceutical companies	Transparency practice, public awareness campaigns, and strengthening regulation [33, 50]

As a result, the research that clarifies and subsequently demonstrates which factors have a significant effect on the variability of antibiotic prescription makes a significant contribution to developing interventions that are efficient and successful.

#### Strengths and limitations

The strength of this review is that we included all study designs to summarize the available evidence. This review solely focused on physicians, and we did not include nurses, pharmacists, caregivers and other healthcare professionals, who may have a role and/or influence in the prescription of antibiotics. These actors often interact closely with patients and have their knowledge, preference and responsibilities regarding antibiotic prescription. Another limitation of this study was that most of the studies were conducted in developed countries, which limits understanding of developing countries where care settings and sociocultural factors may vary. Only studies published in English were reviewed. The included studies used different methodologies leading to methodological heterogeneity, this make it challenging to synthesize findings and draw meaningful comparison.

#### The implication of the results for practice, policy and future research

In this review, we observed that patient-and physicians-related factors contribute significantly to the variation in antibiotic prescription. Implementing patient-centred intervention such as shared decision-making could be effective strategy to reduce this variation [38, 53]. However, this review highlights that there is a need to understand the variability of antibiotic prescriptions between and within physicians. Variation in antibiotic prescribing practices is poorly explained in the included studies, despite justifiable differences in prescription volume. Therefore, confirming the precise reason for the encounter helps reduce the variability of antibiotic prescription in primary health care. Antibiotic prescriptions by allied healthcare professionals, including clinical pharmacists, physician assistants, have increased in primary healthcare settings in recent years [94, 95]. However, their roles were not evaluated in this study, so this needs further investigation.

Furthermore, physicians may prescribe less if management encourages intra-professional discussion within the practice, internalized guidelines, and management of patient expectations across the practice. Moreover, the majority of the studies in this review were carried out in developed nations, indicating the importance of conducting research in a diversity of healthcare settings to understand the contextual factors that affect prescribing and tailoring interventions accordingly.

## Conclusion

In general, variation in antibiotic prescribing among primary health care physicians is explained by several different factors. The major factors that contribute to this variation include physician experience and individual practice patterns, time constraints, physician perceptions and attitudes, colleagues' influence, and patient-related factors (perception and attitudes toward antibiotics). Our review indicates that the level of clinical experience and the use of guidelines counteract the effect of patient expectations on prescribing practices. Variations in antibiotic prescriptions among healthcare professionals in the primary healthcare setting could contribute to increased antibiotic resistance. Thus, studies on the drivers of prescribing habits can guide antibiotic stewardship program efforts. Finally, we suggest that to address factors that influence the variability of antibiotic prescription, interventions should aim to provide continued medical education and training and promote patient-centred care.

### Supplementary Information

**Additional file 1.**

**Additional file 2:** **Table S1.** Appraisal of the methodological quality.

**Additional file 3.**

## Data Availability

All data generated or analysed during this study are included in this manuscript. For any further data, it can be accessible from corresponding author on reasonable request.
