# Supplementary material for: Factors contributing to the variation in antibiotic prescribing among primary health care physicians: a systematic review
Source: BMC Prim Care. 2024 Jan 2;25:8. doi: 10.1186/s12875-023-02223-1 (PMC10759428; doi:10.1186/s12875-023-02223-1)
Supplement: Supplementary file 1 — Additional file 1. [file 12875_2023_2223_MOESM1_ESM.docx]

**Supplementary file 1**

**PubMed search terms**

(("predictor"[All Fields] OR "predictors"[All Fields]) AND ("variabilities"[All Fields] OR "variability"[All Fields] OR "variable"[All Fields] OR "variable s"[All Fields] OR "variables"[All Fields] OR "variably"[All Fields]) AND (("anti bacterial agents"[Pharmacological Action] OR "anti bacterial agents"[MeSH Terms] OR ("anti bacterial"[All Fields] AND "agents"[All Fields]) OR "anti bacterial agents"[All Fields] OR "antibiotic"[All Fields] OR "antibiotics"[All Fields] OR "antibiotic s"[All Fields] OR "antibiotical"[All Fields]) AND ("prescriptions"[MeSH Terms] OR "prescriptions"[All Fields] OR "prescription"[All Fields])) AND ("physician s"[All Fields] OR "physicians"[MeSH Terms] OR "physicians"[All Fields] OR "physician"[All Fields] OR "physicians s"[All Fields]) AND ("primary health care"[MeSH Terms] OR ("primary"[All Fields] AND "health"[All Fields] AND "care"[All Fields]) OR "primary health care"[All Fields] OR ("primary"[All Fields] AND "healthcare"[All Fields]) OR "primary healthcare"[All Fields])) AND ((fha[Filter]) AND (meta-analysis[Filter] OR observationalstudy[Filter] OR randomizedcontrolledtrial[Filter] OR review[Filter] OR systematicreview[Filter]))

**Web sciences:**

Results for (((((TS=("antibiotic prescribing ")) AND TS=(physician* )) OR TS=(doctors*)) AND TS=("primary health care")) AND TS=(factors*)) OR TS=(predictors*) and Review Article (Exclude – Document Types) and Early Access (Exclude – Document Types) and Article (Document Types) and Data Paper or Retracted Publication or Book Chapters or Proceeding Paper (Exclude – Document Types) and Withdrawn Publication or Publication With Expression Of Concern (Exclude – Document Types) and All Open Access (Open Access) and English (Languages) (((ALL=(antibiotic prescribing )) AND ALL=(physicians )) AND ALL=(primary health care)) AND ALL=(factors ) Results for (((ALL=(antibiotic prescribing )) AND ALL=(physicians )) AND ALL=(primary health care)) AND ALL=(factors ) and All Open Access (Open Access) and English (Languages) and 1.23 Antibiotics & Antimicrobials (Citation Topics Meso) Results for antibiotic prescribing (Title) and 2023 or 2022 or 2021 or 2020 or 2019 (Publication Years) and Article (Document Types) and All Open Access (Open Access) and English (Languages)

**Scopus:**

TITLE-ABS-KEY ( predictors AND variability AND of AND antibiotic AND prescribing

AND among AND physicians AND in AND primary AND health AND care ) AND

( LIMIT-TO ( SUBJAREA , "MEDI" ) ) AND ( LIMIT-TO ( DOCTYPE , "ar" ) ) AND ( LIMIT-TO ( LANGUAGE , "English" ) ) AND ( LIMIT-TO ( SRCTYPE , "j" ) ) AND ( LIMIT-TO ( OA , "all" ) )

TITLE-ABS-KEY ( antibiotic* AND physician* AND primary* AND health* ) AND ( EXCLUDE ( OA , "repository" ) OR EXCLUDE ( OA , "publisherfullgold" ) OR EXCLUDE ( OA , "publisherfree2read" ) OR EXCLUDE ( OA , "publisherhybridgold" ) ) AND ( EXCLUDE ( DOCTYPE , "re" ) OR EXCLUDE ( DOCTYPE , "le" ) OR EXCLUDE ( DOCTYPE , "ed" ) OR EXCLUDE ( DOCTYPE , "no" ) ) AND ( EXCLUDE ( SRCTYPE , "b" ) OR EXCLUDE ( SRCTYPE , "k" ) OR EXCLUDE ( SRCTYPE , "Undefined" ) )

Or TITLE-ABS-KEY ( antibiotic* AND prescription* AND physician* AND primary* AND health* ) AND ( LIMIT-TO ( SRCTYPE , "j" ) ) AND ( LIMIT-TO ( OA , "all" ) ) AND ( LIMIT-TO ( PUBSTAGE , "final" ) ) AND ( LIMIT-TO ( DOCTYPE , "ar" ) ) AND ( LIMIT-TO ( LANGUAGE , "English" ) ) AND ( LIMIT-TO ( EXACTKEYWORD , "Article" ) )

**ProQuest health and medicine**

title("antibiotic prescription") AND subject(Physician) AND subject("primary health care") AND subject(predictor OR factors) OR subject(factors)

title(antibiotic prescribing) AND physicians OR doctors AND title(primary health care) AND title(predictors OR factors)
