# Supplementary material for: Factors contributing to the variation in antibiotic prescribing among primary health care physicians: a systematic review
Source: BMC Prim Care. 2024 Jan 2;25:8. doi: 10.1186/s12875-023-02223-1 (PMC10759428; doi:10.1186/s12875-023-02223-1)
Supplement: Supplementary file 2 — Additional file 2: Table S1. Appraisal of the methodological quality. [file 12875_2023_2223_MOESM2_ESM.docx]

**Table S1**: **Appraisal of the methodological quality**

| **Methodological quality criteria** | Palin et al. (2019) | Lum et al. (2018) | Velden et al. (2022) | Theodorou et al. (2009) | Queder et al. (2022) | Chan et al. (2019) | Kotwania et al. (2010) | Fletcher-Lartey et al. (2016) | Laka et al. (2021) | Swe et al. (2020) | Mousque` s et al. (2010) |
| --- | --- | --- | --- | --- | --- | --- | --- | --- | --- | --- | --- |
| Are there clear qualitative and quantitative research questions (or objectives*), or a clear mixed methods question (or objective*)? |  |  |  |  |  |  |  |  |  |  |  |
| Do the collected data allow address the research question (objective)? |  |  |  |  |  |  |  |  |  |  |  |
| **1 Qualitative** | | | | | | | |  |  |  |  |
| 1.1. Is the qualitative approach appropriate to answer the research question? |  |  |  |  |  |  |  |  |  |  |  |
| 1.2. Are the qualitative data collection methods adequate to address the research question? |  |  |  |  |  |  |  |  |  |  |  |
| 1.3. Are the findings adequately derived from the data? |  |  |  |  |  |  | x |  |  |  |  |
| 1.4. Is the interpretation of results sufficiently substantiated by data? |  |  |  |  |  | x |  |  |  |  |  |
| 1.5. Is there coherence between qualitative data sources, collection, analysis and interpretation? |  |  |  |  |  |  |  |  |  |  |  |
| **Quantitative, randomized controlled trials** | | | | | | | |  |  |  |  |
| 2.1. Is randomization appropriately performed? |  |  |  |  |  |  |  |  |  |  |  |
| 2.2. Are the groups comparable at baseline? |  |  |  |  |  |  |  |  |  |  |  |
| 2.3. Are there complete outcome data? |  |  |  |  |  |  |  |  |  |  |  |
| 2.4. Are outcome assessors blinded to the intervention provided? |  |  |  |  |  |  |  |  |  |  |  |
| 2.5 Did the participants adhere to the assigned intervention? |  |  |  |  |  |  |  |  |  |  |  |
| **Quantitative, nonrandomized** | | | | | | | |  |  |  |  |
| 3.1. Are the participants representative of the target population? |  |  |  |  |  |  |  |  |  |  |  |
| 3.2. Are measurements appropriate regarding both the outcome and intervention (or exposure)? |  |  |  |  |  |  |  |  |  |  |  |
| 3.3. Are there complete outcome data? |  |  |  |  |  |  |  |  |  |  |  |
| 3.4. Are the confounders accounted for in the design and analysis? |  |  |  |  |  |  |  |  |  |  |  |
| 3.5. During the study period, is the intervention administered (or exposure occurred) as intended? |  |  |  |  |  |  |  |  |  |  |  |
| **Quantitative, descriptive** | | | | | | | |  |  |  |  |
| 4.1. Is the sampling strategy relevant to address the research question? |  |  |  |  |  |  |  |  |  |  |  |
| 4.2. Is the sample representative of the target population? |  |  |  |  |  |  |  |  |  |  |  |
| 4.3. Are the measurements appropriate? |  |  |  |  |  |  |  |  |  | x |  |
| 4.4. Is the risk of nonresponse bias low? |  |  |  | x |  |  |  |  |  |  | x |
| 4.5. Is the statistical analysis appropriate to answer the research question |  |  |  |  |  |  |  |  |  |  |  |
| **5. Mixed methods** | | | | | | | |  |  |  |  |
| 5.1. Is there an adequate rationale for using a mixed methods design to address the research question? |  |  |  |  |  |  |  |  |  |  |  |
| 5.2. Are the different components of the study effectively integrated to answer the research question? |  |  |  |  |  |  |  |  |  |  |  |
| 5.3. Are the outputs of the integration of qualitative and quantitative components adequately interpreted? |  |  |  |  |  |  |  |  |  |  |  |
| 5.4. Are divergences and inconsistencies between quantitative and qualitative results adequately addressed? |  | x |  |  |  |  |  |  |  |  |  |
| 5.5. Do the different components of the study adhere to the quality criteria of each tradition of the methods involved? |  |  |  |  |  |  |  |  |  |  |  |
|  | | | | | | | |  |  |  |  |
| **Methodological quality criteria** | Cadieux et al. (2007) | Skodvin et al. (2015) | Guo et al. (2021) | Borek et al. (2020) | Be´jean et al. (2007) |  | Schwartz et al. (2019) | Aabenhus et al.(2017) | Pouwels et al. (2018) | Paluck et al. (2001) |  |
| Are there clear qualitative and quantitative research questions (or objectives*), or a clear mixed methods question (or objective*)? |  |  |  |  |  |  |  |  |  |  |  |
| Do the collected data allow address the research question (objective)? |  |  |  |  |  |  |  |  |  |  |  |
| 1. **Qualitative** | | | | | | | |  |  |  |  |
| 1.1. Is the qualitative approach appropriate to answer the research question? |  |  |  |  |  |  |  |  |  |  |  |
| 1.2. Are the qualitative data collection methods adequate to address the research question? |  |  |  |  |  |  |  |  |  |  |  |
| 1.3. Are the findings adequately derived from the data? |  |  |  |  |  |  |  |  |  |  |  |
| 1.4. Is the interpretation of results sufficiently substantiated by data? |  |  |  |  |  |  |  |  |  |  |  |
| 1.5. Is there coherence between qualitative data sources, collection, analysis and interpretation? |  |  |  | x |  |  |  |  |  |  |  |
| **Quantitative, randomized controlled trials** | | | | | | | |  |  |  |  |
| 2.1. Is randomization appropriately performed? |  |  |  |  |  |  |  |  |  |  |  |
| 2.2. Are the groups comparable at baseline? |  |  |  |  |  |  |  |  |  |  |  |
| 2.3. Are there complete outcome data? |  |  |  |  |  |  |  |  |  |  |  |
| 2.4. Are outcome assessors blinded to the intervention provided? |  |  |  |  |  |  |  |  |  |  |  |
| 2.5 Did the participants adhere to the assigned intervention? |  |  |  |  |  |  |  |  |  |  |  |
| **Quantitative, nonrandomized** | | | | | | | |  |  |  |  |
| 3.1. Are the participants representative of the target population? |  |  |  |  |  |  |  |  |  |  |  |
| 3.2. Are measurements appropriate regarding both the outcome and intervention (or exposure)? |  |  |  |  |  |  |  |  |  |  |  |
| 3.3. Are there complete outcome data? |  |  |  |  |  |  |  |  |  |  |  |
| 3.4. Are the confounders accounted for in the design and analysis? |  |  |  |  |  |  |  |  |  |  |  |
| **Quantitative, descriptive** | | | | | | | |  |  |  |  |
| 4.1. Is the sampling strategy relevant to address the research question? |  |  |  |  |  |  |  |  |  |  |  |
| 4.2. Is the sample representative of the target population? |  |  |  |  |  |  |  |  |  |  |  |
| 4.3. Are the measurements appropriate? |  |  |  |  |  |  | x |  |  | x |  |
| 4.4. Is the risk of nonresponse bias low? |  |  |  |  |  |  |  |  |  |  |  |
| 4.5. Is the statistical analysis appropriate to answer the research question |  |  |  |  |  |  | x | x |  |  |  |
| **5. Mixed methods** | | | | | | | |  |  |  |  |
| 5.1. Is there an adequate rationale for using a mixed methods design to address the research question? |  |  |  |  |  |  |  |  |  |  |  |
| 5.2. Are the different components of the study effectively integrated to answer the research question? |  |  |  |  |  |  |  |  |  |  |  |
| 5.3. Are the outputs of the integration of qualitative and quantitative components adequately interpreted? |  |  |  |  |  |  |  |  |  |  |  |
| 5.4. Are divergences and inconsistencies between quantitative and qualitative results adequately addressed? |  |  |  |  |  |  |  |  |  |  |  |
| 5.5. Do the different components of the study adhere to the quality criteria of each tradition of the methods involved? |  |  |  |  |  |  |  |  |  |  |  |
|  | | | | | | | |  |  |  |  |
| **Methodological quality criteria** | Kumar et al.(2003) | Simpson et a.(2007) | Wood et al. (2007) | Reynolds and McKee (2009) | Björkman et al. (2010) | Björkman et al. (2011) |  | Vazquez et al.(2011) |  | Akkerman et al. (2005) | Wester et al (2002) |
| Are there clear qualitative and quantitative research questions (or objectives*), or a clear mixed methods question (or objective*)? |  |  |  |  |  |  |  |  |  |  |  |
| Do the collected data allow address the research question (objective)? |  |  |  |  |  |  |  |  |  |  |  |
| 1. **Qualitative** | | | | | | | |  |  |  |  |
| 1.1. Is the qualitative approach appropriate to answer the research question? |  |  |  |  |  |  |  |  |  |  |  |
| 1.2. Are the qualitative data collection methods adequate to address the research question? | x |  |  |  |  |  |  |  |  |  |  |
| 1.3. Are the findings adequately derived from the data? |  |  |  |  |  |  |  |  |  |  |  |
| 1.4. Is the interpretation of results sufficiently substantiated by data? |  | x |  |  |  |  |  | x |  |  |  |
| 1.5. Is there coherence between qualitative data sources, collection, analysis and interpretation? | x |  | x |  |  |  |  |  |  |  |  |
| **Quantitative, randomized controlled trials** | | | | | | | |  |  |  |  |
| 2.1. Is randomization appropriately performed? |  |  |  |  |  |  |  |  |  |  |  |
| 2.2. Are the groups comparable at baseline? |  |  |  |  |  |  |  |  |  |  |  |
| 2.3. Are there complete outcome data? |  |  |  |  |  |  |  |  |  |  |  |
| 2.4. Are outcome assessors blinded to the intervention provided? |  |  |  |  |  |  |  |  |  |  |  |
| 2.5 Did the participants adhere to the assigned intervention? |  |  |  |  |  |  |  |  |  |  |  |
| **Quantitative, nonrandomized** | | | | | | | |  |  |  |  |
| 3.1. Are the participants representative of the target population? |  |  |  |  |  |  |  |  |  |  |  |
| 3.2. Are measurements appropriate regarding both the outcome and intervention (or exposure)? |  |  |  |  |  |  |  |  |  |  |  |
| 3.3. Are there complete outcome data? |  |  |  |  |  |  |  |  |  |  |  |
| 3.4. Are the confounders accounted for in the design and analysis? |  |  |  |  |  |  |  |  |  |  |  |
| **Quantitative, descriptive** | | | | | | | |  |  |  |  |
| 4.1. Is the sampling strategy relevant to address the research question? |  |  |  |  |  |  |  |  |  |  |  |
| 4.2. Is the sample representative of the target population? |  |  |  |  |  |  |  |  |  |  |  |
| 4.3. Are the measurements appropriate? |  |  |  |  |  |  |  |  |  |  |  |
| 4.4. Is the risk of nonresponse bias low? |  |  |  |  |  |  |  |  |  | x | x |
| 4.5. Is the statistical analysis appropriate to answer the research question |  |  |  |  |  |  |  |  |  |  |  |
| **5. Mixed methods** | | | | | | | |  |  |  |  |
| 5.1. Is there an adequate rationale for using a mixed methods design to address the research question? |  |  |  |  |  |  |  |  |  |  |  |
| 5.2. Are the different components of the study effectively integrated to answer the research question? |  |  |  | x |  |  |  |  |  |  |  |
| 5.3. Are the outputs of the integration of qualitative and quantitative components adequately interpreted? |  |  |  |  | x |  |  |  |  |  |  |
| 5.4. Are divergences and inconsistencies between quantitative and qualitative results adequately addressed? |  |  |  |  |  |  |  |  |  |  |  |
| 5.5. Do the different components of the study adhere to the quality criteria of each tradition of the methods involved? |  |  |  |  |  |  |  |  |  |  |  |
|  | | | | | | | |  |  |  |  |
| **Methodological quality criteria** | van der Zand et al. (2019) | Chem et al. (2018) | Zetts RM et al.(2019) | Rodrigues et al.(2021) | Alradin et al.(2021) | Tang et al. (2021) | Rodrigues et al.(2016) | Al-Homaidan and Barrimah (2018) | Beilfuss et al.(2022) | Frost et al. (2018) | Karimi et al. (2023) |
| Are there clear qualitative and quantitative research questions (or objectives*), or a clear mixed methods question (or objective*)? |  |  |  |  |  |  |  |  |  |  |  |
| Do the collected data allow address the research question (objective)? |  |  |  |  |  |  |  |  |  |  |  |
|  | | | | | | | |  |  |  |  |
| 1.1. Is the qualitative approach appropriate to answer the research question? |  |  |  |  |  |  |  |  |  |  |  |
| 1.2. Are the qualitative data collection methods adequate to address the research question? |  |  |  |  |  |  |  |  |  |  |  |
| 1.3. Are the findings adequately derived from the data? |  |  |  |  |  |  |  |  |  |  |  |
| 1.4. Is the interpretation of results sufficiently substantiated by data? |  |  |  |  |  |  |  |  |  |  |  |
| 1.5. Is there coherence between qualitative data sources, collection, analysis and interpretation? |  |  |  |  | x |  |  |  |  |  |  |
| **Quantitative, randomized controlled trials** | | | | | | | |  |  |  |  |
| 2.1. Is randomization appropriately performed? |  |  |  |  |  |  |  |  |  |  |  |
| 2.2. Are the groups comparable at baseline? |  |  |  |  |  |  |  |  |  |  |  |
| 2.3. Are there complete outcome data? |  |  |  |  |  | x |  |  |  |  |  |
| 2.4. Are outcome assessors blinded to the intervention provided? |  |  |  |  |  |  |  |  |  |  |  |
| 2.5 Did the participants adhere to the assigned intervention? |  |  |  |  |  |  |  |  |  |  |  |
| **Quantitative, nonrandomized** | | | | | | | |  |  |  |  |
| 3.1. Are the participants representative of the target population? |  |  |  |  |  |  |  |  |  |  |  |
| 3.2. Are measurements appropriate regarding both the outcome and intervention (or exposure)? |  |  |  |  |  |  |  |  |  |  |  |
| 3.3. Are there complete outcome data? |  |  |  | ✓ |  |  |  |  |  |  |  |
| 3.4. Are the confounders accounted for in the design and analysis? |  |  |  |  |  |  |  |  |  |  |  |
| **Quantitative, descriptive** | | | | | | | |  |  |  |  |
| 4.1. Is the sampling strategy relevant to address the research question? |  |  |  |  |  |  |  |  |  |  |  |
| 4.2. Is the sample representative of the target population? |  |  |  |  |  |  |  |  |  |  |  |
| 4.3. Are the measurements appropriate? |  |  |  |  |  |  |  |  |  |  |  |
| 4.4. Is the risk of nonresponse bias low? |  | x |  | x | x |  |  | x |  | x |  |
| 4.5. Is the statistical analysis appropriate to answer the research question |  |  |  |  |  | x |  |  |  |  |  |
| **5. Mixed methods** | | | | | | | |  |  |  |  |
| 5.1. Is there an adequate rationale for using a mixed methods design to address the research question? |  |  |  |  |  |  |  |  |  |  |  |
| 5.2. Are the different components of the study effectively integrated to answer the research question? |  |  |  |  |  |  |  |  |  |  |  |
| 5.3. Are the outputs of the integration of qualitative and quantitative components adequately interpreted? |  |  |  |  |  |  |  |  |  |  |  |
| 5.4. Are divergences and inconsistencies between quantitative and qualitative results adequately addressed? |  |  |  |  |  |  |  |  |  |  |  |
| 5.5. Do the different components of the study adhere to the quality criteria of each tradition of the methods involved? |  |  |  |  |  |  |  |  |  |  |  |
|  | | | | | | | |  |  |  |  |
|  |  |  |  |  |  |  |  |  |  |  |  |
|  |  |  |  |  |  |  |  |  |  |  |  |
|  | | | | | | | |  |  |  |  |
| **Methodological quality criteria** | Huang et al. (2021) | Sharaf et al.(2021) | Poss-Doering (2020) | Liu et al.(2019) | Cordoba et al. (2017) | Ahmadi and Zarei (2017) | Bharathiraja et al.(2005) | Bjornsdo´ttir et al.(2010) | Sydenham et al(2022) |  |  |
| Are there clear qualitative and quantitative research questions (or objectives*), or a clear mixed methods question (or objective*)? |  |  |  |  |  |  |  |  |  |  |  |
| Do the collected data allow address the research question (objective)? |  |  |  |  |  |  |  |  |  |  |  |
| 1. **Qualitative** | | | | | | | |  |  |  |  |
| 1.1. Is the qualitative approach appropriate to answer the research question? |  |  |  |  |  |  |  |  |  |  |  |
| 1.2. Are the qualitative data collection methods adequate to address the research question? |  |  |  |  |  |  |  |  |  |  |  |
| 1.3. Are the findings adequately derived from the data? |  |  |  |  |  |  |  | x |  |  |  |
| 1.4. Is the interpretation of results sufficiently substantiated by data? |  | x |  |  |  |  | x |  |  |  |  |
| 1.5. Is there coherence between qualitative data sources, collection, analysis and interpretation? |  | x |  |  |  |  | x | x |  |  |  |
| **Quantitative, randomized controlled trials** | | | | | | | |  |  |  |  |
| 2.1. Is randomization appropriately performed? |  |  |  |  |  |  |  |  |  |  |  |
| 2.2. Are the groups comparable at baseline? |  |  |  |  |  |  |  |  |  |  |  |
| 2.3. Are there complete outcome data? |  |  |  |  |  |  |  |  |  |  |  |
| 2.4. Are outcome assessors blinded to the intervention provided? |  |  |  |  |  |  |  |  |  |  |  |
| 2.5 Did the participants adhere to the assigned intervention? |  |  |  |  |  |  |  |  |  |  |  |
| **Quantitative, nonrandomized** | | | | | | | |  |  |  |  |
| 3.1. Are the participants representative of the target population? |  |  |  |  |  |  |  |  |  |  |  |
| 3.2. Are measurements appropriate regarding both the outcome and intervention (or exposure)? |  |  |  |  |  |  |  |  |  |  |  |
| 3.3. Are there complete outcome data? |  |  |  |  |  |  |  |  |  |  |  |
| 3.4. Are the confounders accounted for in the design and analysis? |  |  |  |  |  |  |  |  |  |  |  |
| **Quantitative, descriptive** | | | | | | | |  |  |  |  |
| 4.1. Is the sampling strategy relevant to address the research question? |  |  |  |  |  |  |  |  |  |  |  |
| 4.2. Is the sample representative of the target population? |  |  |  |  |  |  |  |  |  |  |  |
| 4.3. Are the measurements appropriate? |  |  |  |  |  |  |  |  |  |  |  |
| 4.4. Is the risk of nonresponse bias low? |  |  |  |  | x | x | x |  |  |  |  |
| 4.5. Is the statistical analysis appropriate to answer the research question |  |  |  |  |  |  | x |  |  |  |  |
| **5. Mixed methods** | | | | | | | |  |  |  |  |
| 5.1. Is there an adequate rationale for using a mixed methods design to address the research question? |  |  |  |  |  |  |  |  |  |  |  |
| 5.2. Are the different components of the study effectively integrated to answer the research question? |  |  |  |  |  |  |  |  |  |  |  |
| 5.3. Are the outputs of the integration of qualitative and quantitative components adequately interpreted? |  |  |  |  |  |  |  |  |  |  |  |
| 5.4. Are divergences and inconsistencies between quantitative and qualitative results adequately addressed? | x |  |  |  |  |  |  |  |  |  |  |
| 5.5. Do the different components of the study adhere to the quality criteria of each tradition of the methods involved? |  |  |  |  |  |  |  |  |  |  |  |
|  | | | | | | | |  |  |  |  |

- Note: * = yes; x =no
